# Editorial: Bringing visibility to marginalized populations: neuroscience of aging in older adults facing social vulnerability

**DOI:** 10.3389/fnhum.2026.2007329

**Published:** 2026-10-14

**Authors:** Ana C. Teixeira-Santos, Diana R. Pereira, Lucas Murrins Marques, Anja K. Leist, Shenzhao Lu, Heloísa Gonçalves Ferreira, Joana Carvalheiro

**Affiliations:** 1 Institute for Research on Socio-Economic Inequality, University of Luxembourg, Esch-sur-Alzette, Luxembourg; 2 School of Psychology, University of Minho, Braga, Portugal; 3 Centre for Philosophical and Humanistic Studies, Faculty of Philosophy and Social Sciences, Universidade Católica Portuguesa, Braga, Portugal; 4 Mental Health Department, Santa Casa de Sao Paulo School of Medical Sciences, São Paulo, Brazil; 5 Institute of Gerontology, Heidelberg University, Heidelberg, Germany; 6 Department of Molecular and Human Genetics, Baylor College of Medicine, Houston, TX, United States; 7 Jan and Dan Duncan Neurological Research Institute, Texas Children's Hospital, Houston, TX, United States; 8 Psychology Institute, Rio de Janeiro State University, Rio de Janeiro, Brazil; 9 School of Psychology and Neuroscience, University of Glasgow, Glasgow, United Kingdom

**Keywords:** cognitive aging, cognitive assessment, global aging, health inequalities, inclusive research, marginalized populations, Social Determinants of Health (MeSH), social vulnerability

There is a paradox at the heart of aging neuroscience: the people most burdened by cognitive decline and dementia are, too often, those we know the least about. Most of the world's older population, including an increasing proportion of people living with dementia, resides in low- and middle-income countries. Yet, research on cognitive aging continues to rely predominantly on evidence generated in high-income settings (World Health Organization, 2026). This imbalance will become increasingly consequential, as nearly 80% of older adults are projected to live in these countries by 2050 (World Health Organization, 2025). Additionally, older adults living under conditions of social vulnerability, whether shaped by poverty, racial discrimination, geographic isolation, migration and displacement, political violence, or limited access to education and healthcare, bear a disproportionate share of neurocognitive disorders worldwide, yet remain peripheral figures in most research agendas.

This Research Topic was created with that paradox in mind. “*Bringing visibility to marginalized populations: neuroscience of aging in older adults facing social vulnerability*” brought together researchers committed to studying aging where it is most urgent, not only where it is most convenient. Volume I gathered 11 contributions from multiple countries and disciplinary traditions, each illuminating a different facet of the relationship between social disadvantage and brain health in later life.

## When inequality gets under the skin

Perhaps the clearest through line across this Research Topic is the weight of structural and socioeconomic inequalities on cognitive aging. Across Black and Brown Brazilians and older Latin American migrants, socioeconomic disadvantage and limited educational opportunities were associated with poorer cognitive outcomes and greater dementia risk (Tardelli et al.; Menezes et al.; Ribeiro et al.). These inequalities can emerge strikingly early: in one Brazilian cohort, cognitive decline began almost a decade earlier among Black and Brown adults than among White peers, with socioeconomic disadvantage rather than genetic ancestry better accounting for these disparities in dementia risk (Tardelli et al.). Literacy and other routinely available health and socioeconomic factors also predicted dementia risk, highlighting opportunities for low-cost identification in resource-limited settings (Menezes et al.).

## Social adversity and disconnection

Several contributions extend this focus from structural inequalities to the lived experience of social adversity. Among conflict-affected adults in Colombia, exposure to violence was associated with poorer executive function, while educational attainment emerged as an important protective factor (Camargo et al.). Other studies highlight the consequences of social disconnection in later life. Among older adults in China, social alienation was associated with poorer psychological and cognitive outcomes, while meaningful intergenerational communication emerged as potential sources of protection (Sun et al.; Xia et al.). More broadly, loneliness and social isolation were associated with changes in language functioning (Araújo et al.), suggesting that linguistic markers may provide a promising but understudied window into social disconnection. Together, these studies illustrate how different forms of social adversity, from violence to social disconnection, may become embedded in cognitive and psychological aging, while also identifying potential sources of resilience.

## The measurement problem

Running through several contributions in this volume is an uncomfortable question: are our cognitive assessments measuring what we think they do in socially vulnerable populations? Tools developed and validated in highly educated populations may perform poorly, or become unusable, in different social and educational contexts. In Brazil, a widely used cognitive screening tool showed poor accuracy among individuals with low educational attainment (Pereira et al.), while in conflict-affected communities in Colombia, the Stroop Test had to be abandoned because more than half of participants could not complete it due to reading difficulties (Camargo et al.). Such cases illustrate a broader problem of data inequity, whereby barriers in recruitment, measurement, and analysis can systematically exclude disadvantaged populations and limit generalizability (Leist et al.). Greater representation alone is therefore insufficient; research methods must also be appropriate for the populations they investigate.

## Clinical complexity and cognitive vulnerability

Vulnerability in later life is not exclusively social: clinical complexity can also increase susceptibility to cognitive decline. These contributions highlight how acute physiological challenges and accumulated health burdens may compound cognitive vulnerability. In older surgical patients, cerebral oxygen desaturation was associated with adverse neurocognitive outcomes, although evidence that near-infrared spectroscopy (NIRS)-guided interventions can prevent these complications remains inconclusive (Meng et al.). Among older adults living with HIV and multimorbidity in a Chinese sample, nearly half screened positive for mild cognitive impairment, with greater comorbidity burden emerging as an important predictor, whereas education, social support, and self-management were associated with lower risk (Ge et al.).

## What this volume leaves us with

Read together, these 11 contributions reveal multiple, interacting pathways to cognitive vulnerability (Figure 1). Education appears repeatedly, not merely as a demographic variable but as a marker of cognitive reserve and access to resources that may buffer against adversity. More broadly, socioeconomic disadvantage, racial and ethnic inequalities, migration, violence, social disconnection, and clinical complexity can accumulate and interact across the life course, challenging single-risk explanations of cognitive aging and reinforcing the need for multidimensional approaches to risk assessment, prevention, and care.

**Figure 1:**
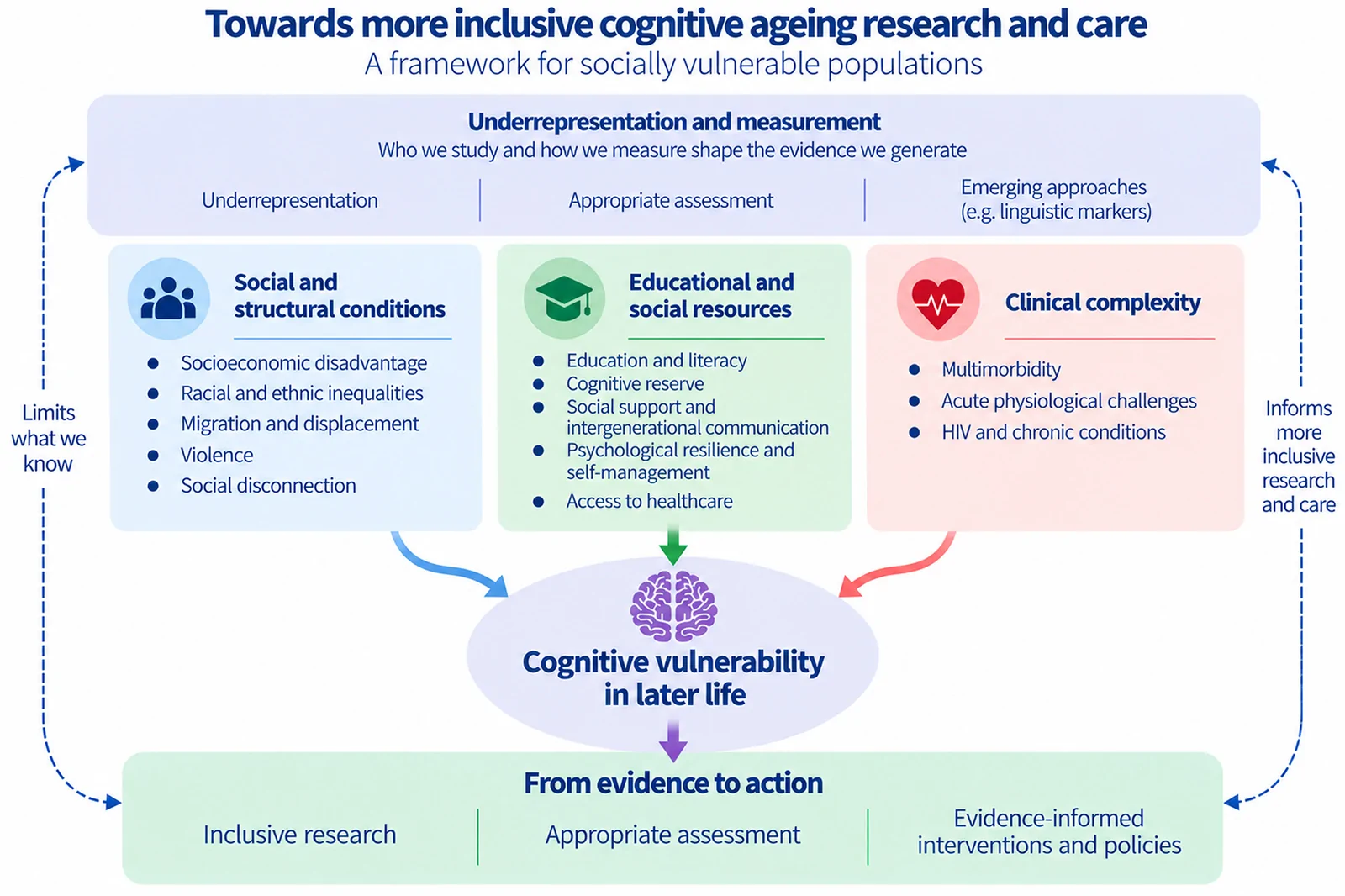
Overview of the key pathways to cognitive vulnerability highlighted across the contributions to this Research Topic.

At the same time, the volume exposes limitations in whose cognitive aging we study and how we study it. Populations carrying a disproportionate burden of cognitive risk remain underrepresented, while standard assessment tools may conflate educational or cultural differences with cognitive impairment. Addressing data inequity therefore requires not only broader representation, but also assessment tools, study designs, and analytical approaches appropriate for diverse social, cultural, and educational contexts (Leist et al.).

These gaps define a research agenda. Volume II provides an opportunity to further expand the representation of populations still peripheral to aging research, strengthen culturally and educationally appropriate measurement, and pursue deeper mechanistic and psychosocial questions about aging under conditions of vulnerability. Longitudinal and methodologically inclusive approaches will be essential to inform interventions and policies that adequately respond to this growing body of evidence.
